# Supplementary figures and images for: Association between pet ownership and physical function at discharge in hospitalized older adults: A retrospective observational study
Source: PLoS One. 2025 Aug 12;20(8):e0330378. doi: 10.1371/journal.pone.0330378 (PMC12342327; doi:10.1371/journal.pone.0330378)

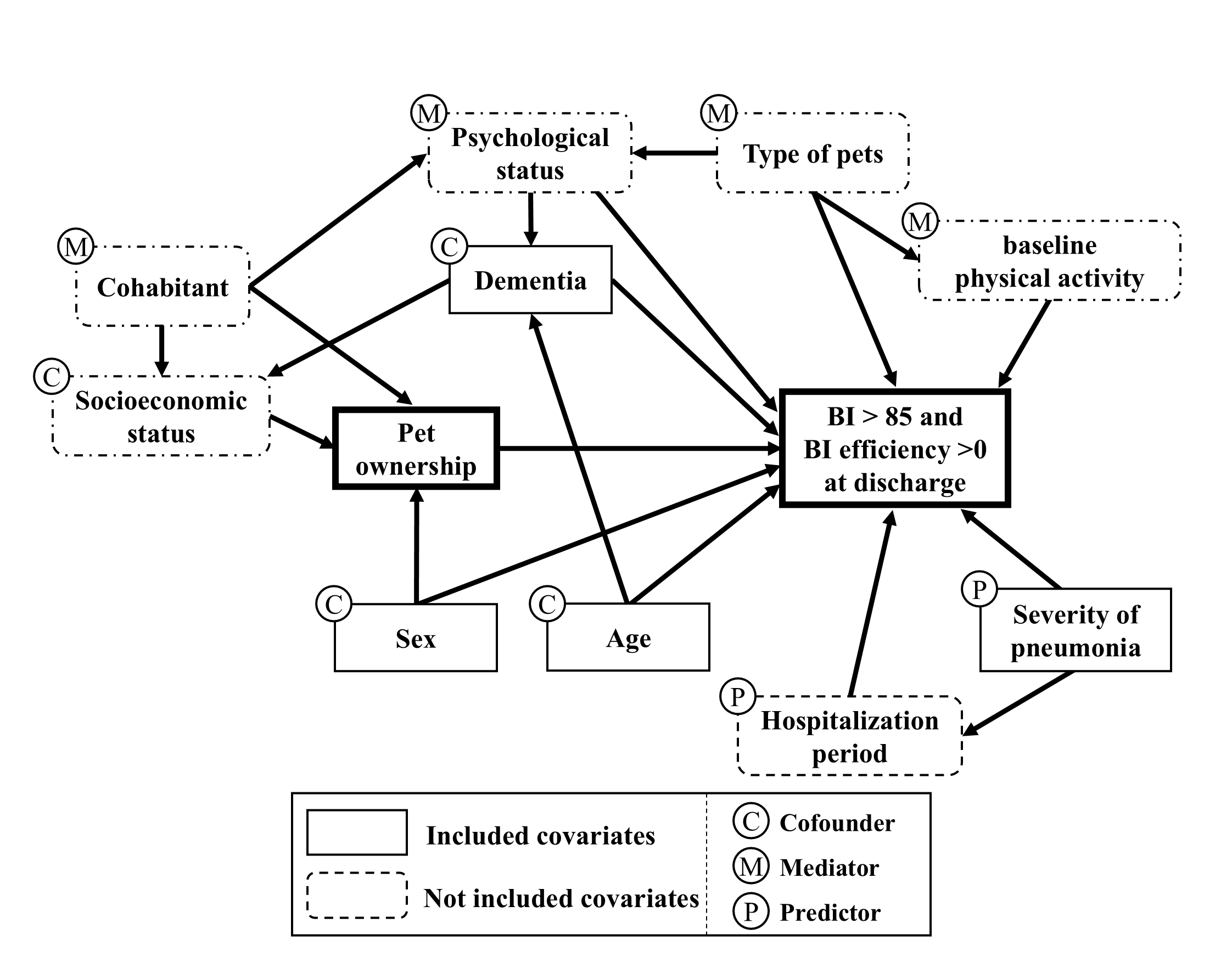

Supplement: S1 Fig — Directed Acyclic Graph of the study. (TIF) [file pone.0330378.s001.tif]
