# Supplementary material for: Association between pet ownership and physical function at discharge in hospitalized older adults: A retrospective observational study
Source: PLoS One. 2025 Aug 12;20(8):e0330378. doi: 10.1371/journal.pone.0330378 (PMC12342327; doi:10.1371/journal.pone.0330378)
Supplement: S1 Table — This model illustrates the total effect of pet ownership on the outcome, without conditioning on PSI, which lies outside the causal pathway according to the assumptions of the Directed Acyclic Graph. (DOCX) [file pone.0330378.s002.docx]

**S1 Table. Logistic regression analysis without adjustment for Pneumonia Severity Index (PSI)**

This model illustrates the total effect of pet ownership on the outcome, without conditioning on PSI, which lies outside the causal pathway according to the assumptions of the Directed Acyclic Graph (S1 Fig).

|  | **Adjusted odds ratio (95% confidential interval)** | ***p* value** |
| --- | --- | --- |
| **Pet ownership** | 0.70 (0.16-4.08) | 0.66 |
