# Supplementary material for: Association between pet ownership and physical function at discharge in hospitalized older adults: A retrospective observational study
Source: PLoS One. 2025 Aug 12;20(8):e0330378. doi: 10.1371/journal.pone.0330378 (PMC12342327; doi:10.1371/journal.pone.0330378)
Supplement: Attachment — Submitted filename: Responce to Reviewers.docx [file pone.0330378.s004.docx]

Dear Dr. Emily Chenette

Thank you for your ongoing consideration of our manuscript for publication in PLOS One. We are grateful for the time and effort you and the reviewers have dedicated to this process. We believe the revised manuscript has been improved. Below, we have addressed the comments provided by you and the reviewers.

We look forward to your editorial decision.

Sincerely,

Yo Ishihara, MD

Department of Palliative Medicine, International University of Health and Welfare Narita Hospital

852, Hatakeda, Narita, Chiba, 286-0124, Japan

TEL: +81-476-35-5600

FAX: +81-476-35-5586

Email: yo-ishihara@iuhw.ac.jp

**************************************************

**Editor:**

**1. Please indicate the grade of pneumonia (mild, moderate or severe) since it can affect the level of physical activity.**
[Response]

Thank you for your valuable comment. We have re-examined the electronic records of all patients, reassigned the Pneumonia Severity Index (PSI) scores, and presented the distribution by class I–V in the revised Table 1. Furthermore, in the logistic regression analysis, we have changed the adjustment factor for severity from ambulance use to PSI.

**2. Please indicate the method of pneumonia treatment as it also affect the level of physical activity. Did any patient receive mechanical ventilation,...etc**
[Response]

Thank you for your comment. We have summarized the data on patients who received mechanical ventilation and included it in the revised Table 1.

**3. There is a major limitation here that could turn your results to be insignificant which is the comorbidity and the elder age. Many patients had stroke, malignancy, even severe cases of pneumonia,..etc. Those patients would have low physical activity whether they own pets or no. Please elaborate in discussion and also explain this in conclusion for future studies as this is a strong limitation in your study.**

[Response]

Thank you for your comment. We have added the following statement to the Discussion section of the revised manuscript on **Lines 270-275**. “Furthermore, the severity of stroke or malignancy may have contributed to a lower baseline physical function. As this was an observational study based solely on electronic medical record data, information on social participation, employment status, psychological condition, and physical impairments could not be collected unless documented, and such details were rarely available. This represents a major limitation of this study.”

**4. Rewrite the conclusion since it is firm while you had many limitations that would turn the results upside down if a new study have addressed them well.**

[Response]

Thank you for your comment. We have revised the conclusion of the study as follows.

**Lines 279-285**: “In conclusion, the present study found no evidence that pet ownership at home is associated with improved physical function during hospitalization or greater independence in ADLs at discharge among older adults hospitalized for pneumonia. However, several limitations, including potential selection bias, may have influenced the results. Further research is required to clarify the extent of the impact of pet ownership and explore methods to further enhance physical function in hospitalized older adults to improve their quality of life.”

**Reviewer: 2**

**1. INTRODUCTION: Please add more information about Barthel index**

[Response]

Thank you for your valuable suggestions. We have added information about the Barthel index on **lines 66-71** in the Introduction section as follows: “BI is an indicator of physical activity, and a clinical tool used to assess the ability to perform basic activities of daily living (ADLs) [8]. BI score below 85 indicates a need for assistance with ADLs [9], while a BI score above 85 suggests the patient is generally independent but may require minimal assistance [10].”

**2. METHOD: The authors report that they searched electronic medical records using Japanese terms such as “pet,” “dog,” “cat,” “ownership,” and “keeping,” in combination with the insurance disease name “pneumonia” to identify eligible patients. However, this approach raises some concerns regarding the reliability and reproducibility of case identification. Specifically, Japan employs a standardized coding system (e.g., ICD-10 codes) in its national health insurance claims data, which should provide a more accurate and systematic method for identifying cases of pneumonia. It is unclear why the authors chose to rely on keyword searches in the free text or insurance disease name fields rather than using established diagnostic codes. Using text-based searches may increase the risk of selection bias or misclassification, particularly if terminology varies among physicians. Moreover, this method could miss cases that are coded appropriately but do not contain the exact keywords used, or could include cases that mention the keywords without confirming a true diagnosis. To enhance methodological rigor and reproducibility, the authors should justify this choice and, if possible, demonstrate the validity of their text-based approach compared to standard coding methods. Moreover, BI scores were recorded based on physical therapists’ notes, but it does not mention whether these assessments were standardized or validated across therapists. This raises concerns about inter-rater reliability.**

[Response]

Thank you for your valuable comment. Patients were included if pneumonia was detected as the principal diagnosis, the diagnosis leading to hospitalization, or the diagnosis requiring the greatest medical resources. For these cases, we reviewed all inpatient electronic medical records, including imaging findings, and board-certified physicians who had completed specialized training in internal medicine assessed whether the diagnosis of pneumonia was appropriate. Therefore, cases in which pneumonia was not truly diagnosed despite the presence of keywords were not included in the study. Conversely, there was a possibility of missing cases that did not contain the specified keywords, and we recognize this as a limitation of our study. It was not feasible to collect information regarding pet ownership through methods other than keyword searches within the hospital’s electronic medical record system. BI scores were recorded based on the notes written by physical therapists in the electronic medical records. Although it is extremely difficult to verify whether the assessments were standardized across different evaluators based on the available records, it is certain that all rehabilitation interventions were performed by licensed physical therapists and that the BI scores were documented based on their professional knowledge and experience.

**2. METHOD: 99-106: Why the author choose specific comorbidities and omit others like chronic kidney disease or heart failure, Also, psychiatric disease should be specified as this term is too broad (depression, anxiety and so on)**

[Response]

Thank you for your valuable comment. We have reclassified the comorbidities as dementia, schizophrenia, and depression and revised the Methods section accordingly. As you pointed out, chronic kidney disease and heart failure are also likely to affect ADLs; therefore, we have added these comorbidities as well. All of these variables have been included in the revised Table 1.

**3. METHOD: The DAG may omit key confounders that could affect both pet ownership and physical function. For example, socioeconomic status, baseline physical activity, or cognitive function might influence both exposure and outcome. Additionally, as a reader, I would appreciate a clearer explanation of the rationale behind the selection of adjustment variables. For example, it is unclear why psychological status was not included—was it considered a collider or irrelevant in the causal pathway? Including this type of justification would strengthen the methodological transparency. It would also be helpful if the DAGs were labeled to indicate the role of each variable (e.g., confounder, mediator, collider), which would make it easier to understand the assumptions guiding the adjustment strategy.**

[Response]

Thank you for your valuable comment. We have revised the DAG by adding markers to clarify the roles of confounders and mediators. We have also explicitly described the rationale for selecting confounders in the Methods section of the revised manuscript. Regarding psychological status, it was not included because information on psychological conditions could not be obtained from the electronic medical records.

**4. METHOD: 144-156: It is important to note that gender and sex represent distinct concepts; in the DAGs, gender was depicted, while the Methods section reported on sex.**

[Response]

Thank you for your suggestions. We have revised the DAG by replacing “gender” with “sex.”

**5. RESULTS: 196: While the authors present main regression outcome in the results section, they do not provide the complete regression table, including all covariates, their coefficients, confidence intervals, and p-values.**

[Response]

Thank you for your valuable comment. We have now provided the regression coefficients, confidence intervals, and p-values for all covariates in Table 3.

**6．DISCUSSION: 213-219: 252-254: why it is difficult to include those variables ?　Figure 2 shows that 62 patients were excluded from the analysis due to the absence of rehabilitation records. While this criterion is understandable for ensuring data completeness, such an exclusion could introduce selection bias if the lack of rehabilitation records is not random. For example, patients without rehabilitation records may differ systematically from those with records in terms of disease severity, functional status, or access to care. This could potentially bias the results and affect the generalizability of the findings. The authors should acknowledge this as a limitation in the discussion section. Additionally, they could provide a brief comparison of baseline characteristics between included and excluded patients to assess the potential for bias.**

[Response]

Thank you for your valuable comment. Because the absence of rehabilitation records prevented evaluation of the outcome, patients without rehabilitation records were excluded from the study. After reanalyzing the data, we found that 59 patients lacked rehabilitation records. Figure 1 has been revised accordingly. The absence of rehabilitation records likely indicates that no rehabilitation referral was made by the attending physician; however, whether this process was random could not be determined. Given the potential for selection bias, we compared the characteristics of patients without rehabilitation records and those who participated in the study, as presented in the S2 Table. The results showed that patients without rehabilitation records had a significantly lower proportion of men and were significantly younger. Additionally, the prevalence of prior stroke was significantly lower among patients without rehabilitation records, whereas no significant differences were observed between the groups regarding other comorbidities or pneumonia severity. As age, sex, and history of stroke are factors that can influence rehabilitation outcomes and prognosis, these findings suggest the presence of selection bias in the present study.
