# Supplementary material for: Association between pet ownership and physical function at discharge in hospitalized older adults: A retrospective observational study
Source: PLoS One. 2025 Aug 12;20(8):e0330378. doi: 10.1371/journal.pone.0330378 (PMC12342327; doi:10.1371/journal.pone.0330378)
Supplement: Attachment — Submitted filename: Responce_to_Reviewers_auresp_2.docx [file pone.0330378.s005.docx]

Dear Dr. Emily Chenette

Thank you very much for your continued consideration of our manuscript submitted to PLOS One. We sincerely appreciate the time and effort that you and the reviewers have devoted to evaluating our work. We believe that the revisions have substantially improved the quality of the manuscript. Please find below our detailed responses to each of the comments raised by the reviewers. We respectfully await your editorial decision.

Sincerely,

Yo Ishihara, MD

Department of Palliative Medicine, International University of Health and Welfare Narita Hospital

852, Hatakeda, Narita, Chiba, 286-0124, Japan

TEL: +81-476-35-5600

FAX: +81-476-35-5586

Email: yo-ishihara@iuhw.ac.jp

**************************************************

**Revisions**

1. We made minor revisions to the English expressions throughout the manuscript.
2. The affiliation of the first author has been partially revised.
3. We revised the Participants section as follows: “We included patients aged ≥65 years who were hospitalized for pneumonia and whose medical records at the time of admission contained terms related to pet ownership.”(**Lines 84-86**)
4. We added the following sentence to the Data Collection section: “We initially identified electronic medical records of patients whose admission charts contained the following Japanese terms; “pet,” “dog,” “cat,” “ownership,” and “keeping,” and who were coded with a diagnosis of pneumonia under the insurance disease classification system.” **(Lines 89-92)**
5. We added “Dementia affects both socioeconomic status and physical function, and socioeconomic status influences current pet-ownership.” **(Lines 133-135)**
6. We added “Other pets included Japanese rice fish (medaka), ferrets, turtles, goldfish, birds (such as parakeets, Java sparrows, finches, and pigeons), rabbits, hamsters, squirrels, guinea pigs, and armadillo,” because the revised exclusion criteria resulted in an increased number of eligible patients. **(Lines 177-179)**
7. We added “Even after adjusting for age, sex, dementia, and PSI more than class IV as covariates, pet ownership was not associated with improved BI scores and functional independence at discharge.” **(Lines 212-214)**
8. We also revised the results in Table 3. Since our primary aim was to estimate the causal effect of pet ownership on Barthel Index at discharge, we believe that the odds ratios and confidence intervals of other covariates are not interpretable and thus do not warrant inclusion. **(Table 2)**
9. The order of References [11] and [12] has been corrected to match their order of first citation in the manuscript.
10. We revised the Exposure section as follows: “The primary exposure was pet ownership at the time of hospitalization. Patients were categorized into two groups: Pet Owner (PO) group if they had a pet at the time of admission, and Non-Pet Owner (NPO) group if they did not. Among current pet owners, additional categorizations were made based on the pet type, particularly focusing on dog ownership.” **(Lines 110-113)**
11. We added “Socioeconomic status, baseline physical activity, and cognitive function could theoretically act as confounders, as they may influence both pet ownership and physical function on DAG. However, these variables were not consistently documented in the electronic medical records and could not be reliably incorporated into the analysis. Proxy information for these variables was also not available in the electronic medical records.” **(Lines 275-280)**
12. We added logistic regression analysis of BI gain>0 and BI index at discharge>85 according to Dog ownership. **(Table 5)**

**Journal Requirements:**

**1. Please review your reference list to ensure that it is complete and correct. If you have cited papers that have been retracted, please include the rationale for doing so in the manuscript text, or remove these references and replace them with relevant current references. Any changes to the reference list should be mentioned in the rebuttal letter that accompanies your revised manuscript. If you need to cite a retracted article, indicate the article’s retracted status in the References list and also include a citation and full reference for the retraction notice.**

[Response]

We have removed the previous reference:

12. W L, Vde M, RL, WB, NK, GT, Town GI, et al. Defining community acquired pneumonia severity on presentation to hospital: An international derivation and validation study. Thorax. 2003;58: 377–382. doi: 10.1136/thorax.58.5.377.

and replaced it with the following reference:

12. Fine MJ, Auble TE, Yealy DM, Hanusa BH, Weissfeld LA, Singer DE, et al. A prediction rule to identify low-risk patients with community-acquired pneumonia. N Engl J Med. 1997;336(4):243–250. doi: 10.1056/NEJM199701233360402.

This change was made because we re-collected the data using the Pneumonia Severity Index (PSI) instead of the CURB-65 score to assess pneumonia severity in our study.

**Reviewer: 2**

**For comment "METHOD: The DAG may omit key confounders that could affect both pet ownership and physical function. For example, socioeconomic status, baseline physical activity, or cognitive function might influence both exposure and outcome. Additionally, as a reader, I would appreciate a clearer explanation of the rationale behind the selection of adjustment variables. For example, it is unclear why psychological status was not included—was it considered a collider or irrelevant in the causal pathway? Including this type of justification would strengthen the**

**methodological transparency. It would also be helpful if the DAGs were labeled to**

**indicate the role of each variable (e.g., confounder, mediator, collider), which would**

**make it easier to understand the assumptions guiding the adjustment strategy."**

**While I appreciate the inclusion of labeled DAGs and partial rationale in the Methods section, the justification for selecting only three adjustment variables remains insufficient. Psychological status was excluded due to lack of data, rather than a theoretical justification based on the causal framework. Additionally, including the Pneumonia Severity Index (PSI) as an adjustment variable—despite acknowledging it may act as a mediator—raises concerns about potential bias from conditioning on a variable in the causal pathway. The rationale for estimating a direct versus total effect is not discussed. I encourage the authors to:**

**Justify the inclusion of PSI under this estimand;**

**More comprehensively discuss why other plausible confounders (e.g., SES, baseline physical activity, cognitive function) were excluded;**

**If model complexity was a constraint, please report the sample size and modeling approach used to evaluate overfitting.**

[Response]

We sincerely appreciate the reviewer’s insightful comments. “PSI” is not considered to be associated with pet ownership; however, it is regarded as a strong prognostic factor that significantly influences the outcome. The primary objective of our study was to estimate the controlled direct effect of pet ownership on the outcome, and thus we included “PSI” as a covariate in the analysis. Although the results remained similar, we also conducted a sensitivity analysis excluding “PSI” to evaluate the total effect, which is presented in S1 Table. “Age” and “sex” were considered confounding factors. “Dementia” was also included in the analysis as a confounder, as it may be influenced by “socioeconomic status” (which affects pet ownership) and may also impact the outcome. While we acknowledge that variables such as “socioeconomic status”, “baseline physical activity” and “psychological status” could potentially serve as important covariates, we were unable to include them in the analysis due to a lack of consistent data. Our analysis was based on data carefully extracted from electronic medical records, and variables not recorded in the records could not be incorporated. We recognize this as a limitation of our study. However, these were not consistently recorded in the electronic medical records and could not be incorporated into the model in a reliable and systematic manner.

We have clarified these points in the revised Methods section **(Lines 131-147)** and Discussion section **(Lines 254-285).**

**Reviewer: 3**

**Lines 139-140: you mentioned that Pneumonia severity may act as a mediator and a previous statement is that Pet ownership is unlikely to influence the severity of Pneumonia. A mediator should be in the middle of the pathway between ethe exposure and the outcome. If Pneumonia was not caused by pet ownership it cant be a mediator. However, you can still adjust for it if it causes the outcome; so no need to change the analysis.**

[Response]

Thank you very much for your insightful comment regarding the role of PSI in our causal framework. In our study, we assumed that pet ownership and pneumonia severity (as measured by the Pneumonia Severity Index, PSI) are not causally related. However, because PSI is a strong prognostic factor for functional outcomes such as the Barthel Index (BI), we included it in the model as a precision variable to improve the accuracy of the estimated association. Our analysis was intended to estimate the direct effect of pet ownership on physical function, not the total effect that may be mediated through pneumonia severity. However, to also examine the total effect of pet ownership, we have added the corresponding results in **S1 Table**. To reflect this more clearly, we revised **Lines 138-141** as follows: ”Although pneumonia severity, as reflected by PSI, is unlikely to be affected by pet ownership, it may significantly influence physical function outcomes. Pneumonia severity index may act as a prognostic factor, so we included the PSI as an adjustment variable to examine the direct effect of pet ownership on physical function.”

**Fig1: any Inclusion or exclusion that happens on future events after the exposure can cause "Immortal time Bias"; lacking records of pet ownership and being hospitalized for the first time happens at the admission. Records missing should be treated as data missing not as an exclusion criteria. Patients that died during hospitalization should still be included and then censored at death. Please update the graph and the analysis accordingly. The graph should have no conditioning on future events (i.e. no inclusion, or exclusion criteria on the follow up period), and the characteristics table (the analysis) should include patients who owns pets upon admission, later on, if death occurs, they may be censored. Meaning they should be added to the baseline variables calculations, and then censored at the outcome.**

[Response]

Thank you very much for your insightful feedback. After re-examining our approach, we fully agree with your assessment. In response, we revised our analysis to include all patients regardless of missing pet information, missing rehabilitation records, or in-hospital death, as these should not be treated as exclusion criteria. Consequently, we revised the **Fig.1** accordingly, and updates to **Tables 1**

**Line 84-86** ” We included patients aged ≥65 years who were hospitalized for pneumonia and whose medical records at the time of admission contained terms related to pet ownership.”
